# A Pathologist’s Dilemma: Placental Site Tumor and Its Differential Diagnosis

**DOI:** 10.7759/cureus.79599

**Published:** 2025-02-24

**Authors:** Chaimae Daoudi, Anass Haloui, Nada Akouh, Karich Nassira, Amal Bennani

**Affiliations:** 1 Department of Pathology, Mohammed VI University Hospital, Faculty of Medicine, Mohammed First University, Oujda, MAR

**Keywords:** choriocarcinoma, cytotrophoblast, epitheloid trophoblastic tumor, exaggerated placental site, implantation site intermediate trophoblast, placental site nodule, placental site trophoblastic tumor, syncytiotrophoblast

## Abstract

Placental site trophoblastic tumor (PSTT) is a rare neoplasm originating from intermediate trophoblastic cells at the site of placental implantation. Its diagnosis poses significant challenges, particularly in distinguishing it from other gestational trophoblastic tumors, such as epithelioid trophoblastic tumors, choriocarcinoma, and placental site nodules, as well as the benign exaggerated placental site.

We present a case of a 29-year-old woman with persistent gestational trophoblastic disease following a complete hydatidiform mole. A detailed review of the differential diagnoses is provided in tables to aid in the diagnostic process, allowing for a clearer comparison and better understanding of these conditions.

This report highlights the importance of accurately identifying PSTT by eliminating potential differential diagnoses through careful pathological and clinical evaluation. We aim to assist pathologists and clinicians in recognizing this rare condition to ensure timely and appropriate management.

## Introduction

Placental site trophoblastic tumor (PSTT) is a rare neoplasm that constitutes approximately 1-2% of all gestational trophoblastic tumors, with an incidence estimated at one to five cases per 100,000 pregnancies [1,2]. It comprises neoplastic implantation site intermediate trophoblastic cells [3]. Some researchers propose that this tumor originates from neoplastic cytotrophoblastic cells, which subsequently differentiate into the intermediate trophoblastic cells characteristic of the implantation site [4].

First described in 1895, this tumor was initially referred to as “atypical chorioepithelioma.” Over time, its understanding evolved, and it was reclassified under its current terminology to reflect its unique histological and biological characteristics better [3].

PSTTs are distinct from other types of gestational trophoblastic diseases due to their clinical behavior and cellular origins, necessitating specific diagnostic and therapeutic approaches [5].

This article provides an in-depth analysis of the clinical, macroscopic, histological, and immunohistochemical aspects of PSTT and its differential diagnoses, beginning with a case report.

## Case presentation

We report a case of a 29-year-old woman (G3P2) diagnosed with a hydatidiform mole in 2021. She underwent two aspirations; pathological results favored a complete hydatidiform mole. Persistent metrorrhagia following aspirations led to a third procedure, which confirmed the diagnosis of a persistent hydatidiform mole.

Initially, the β-human chorionic gonadotropin (β-hCG) level was 145 mIU/mL, and the patient began chemotherapy with methotrexate, which resulted in the negativization of the β-hCG level (3 mIU/mL).

However, one year later, her β-hCG levels began to rise (155 mUI/ml), leading to a fourth aspiration, which was initially misdiagnosed as a pregnancy. However, the pathology results did not show evidence of pregnancy or any signs of a mole.

**Figure 1:**
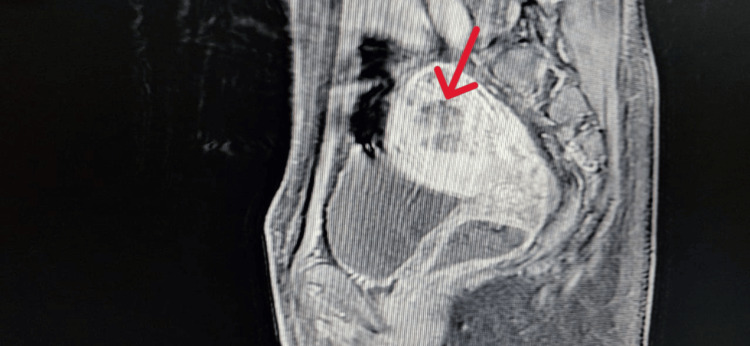
MRI sagittal T1-weighted image showing a globular uterus with a posterior and left lateral corporeal multilocular mass that is poorly defined and hypointense (red arrow).

**Figure 2:**
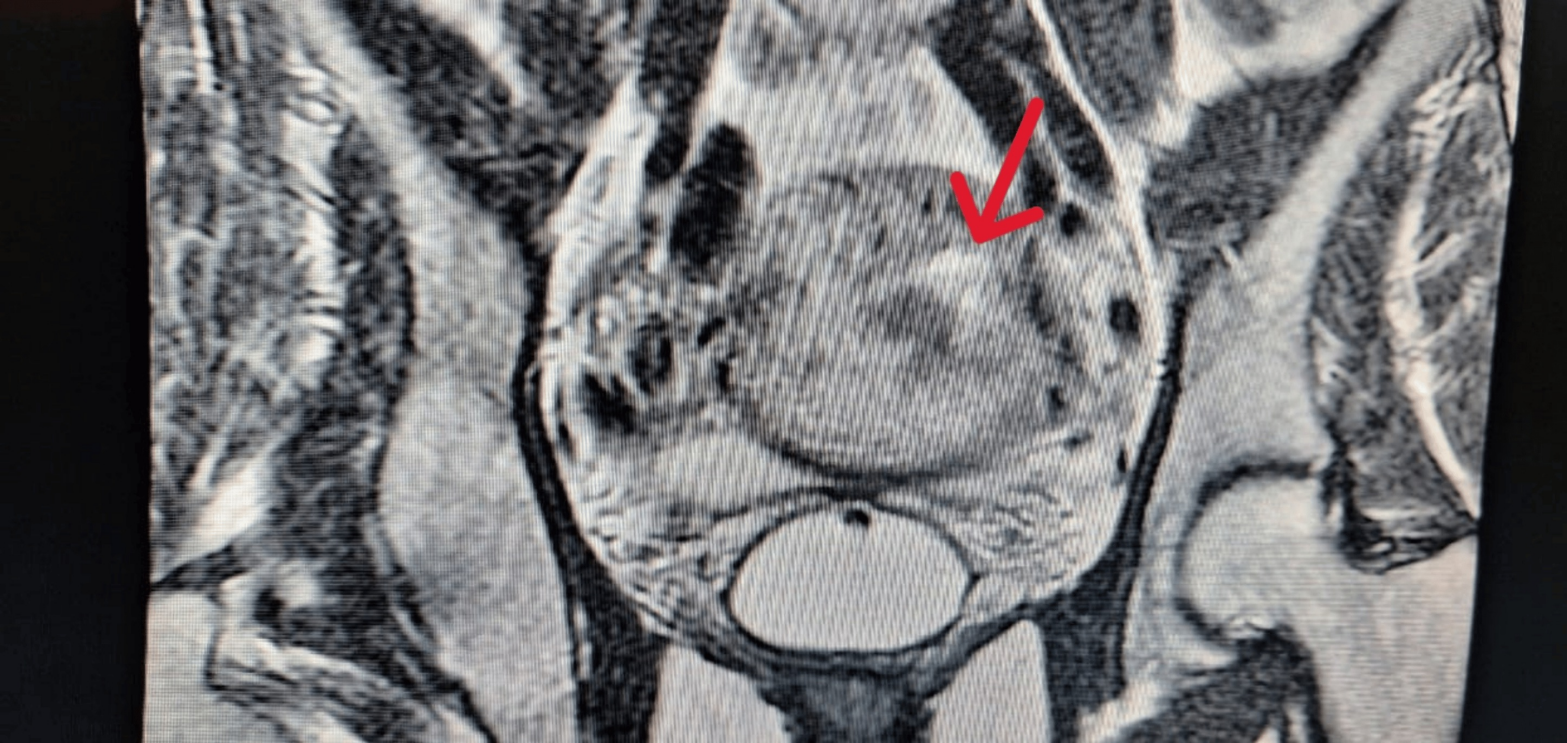
MRI coronal T2-weighted image showing heterogeneous hyperintensity of the mass (red arrow).

The patient underwent a pelvic MRI, which was suggestive of persistent trophoblastic disease (Figures 1, 2).

She was then referred to oncology, where she underwent a polychemotherapy protocol (etoposide, methotrexate, actinomycin D, cyclophosphamide, vincristine (EMA-CO)) consisting of six cycles, with the last cycle administered on December 20, 2023. Subsequent monitoring showed a decrease in β-hCG levels (3.81 mUI/ml), followed by a plateau (5.6-9 mUI/ml).

**Figure 3:**
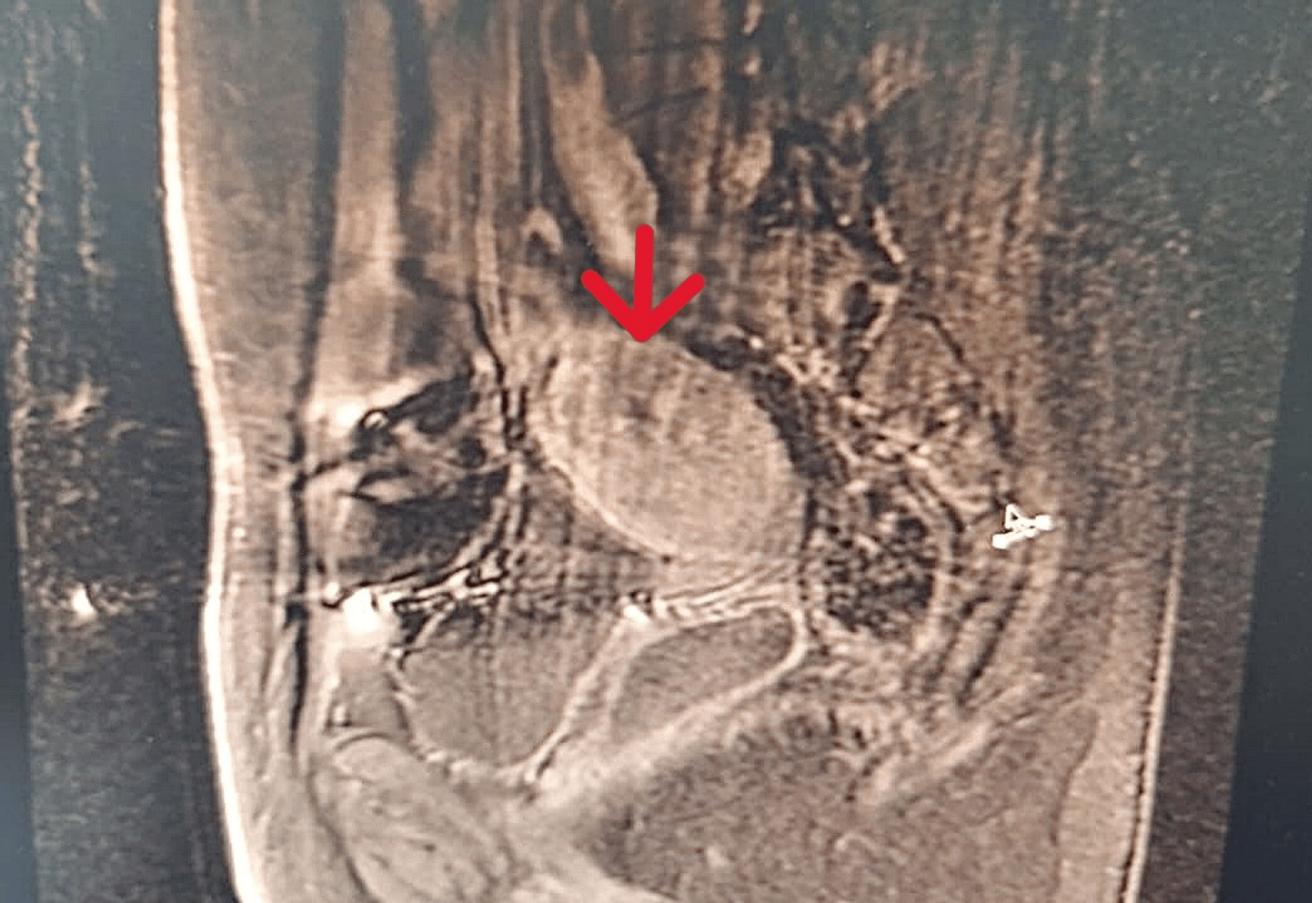
Radiological image revealing the persistence of the lesion process after chemotherapy (red arrow).

The follow-up MRI showed near-stability of the lesion process infiltrating the myometrium, consistent with persistent trophoblastic disease, leading to the decision to perform a hysterectomy (Figure 3).

**Figure 4:**
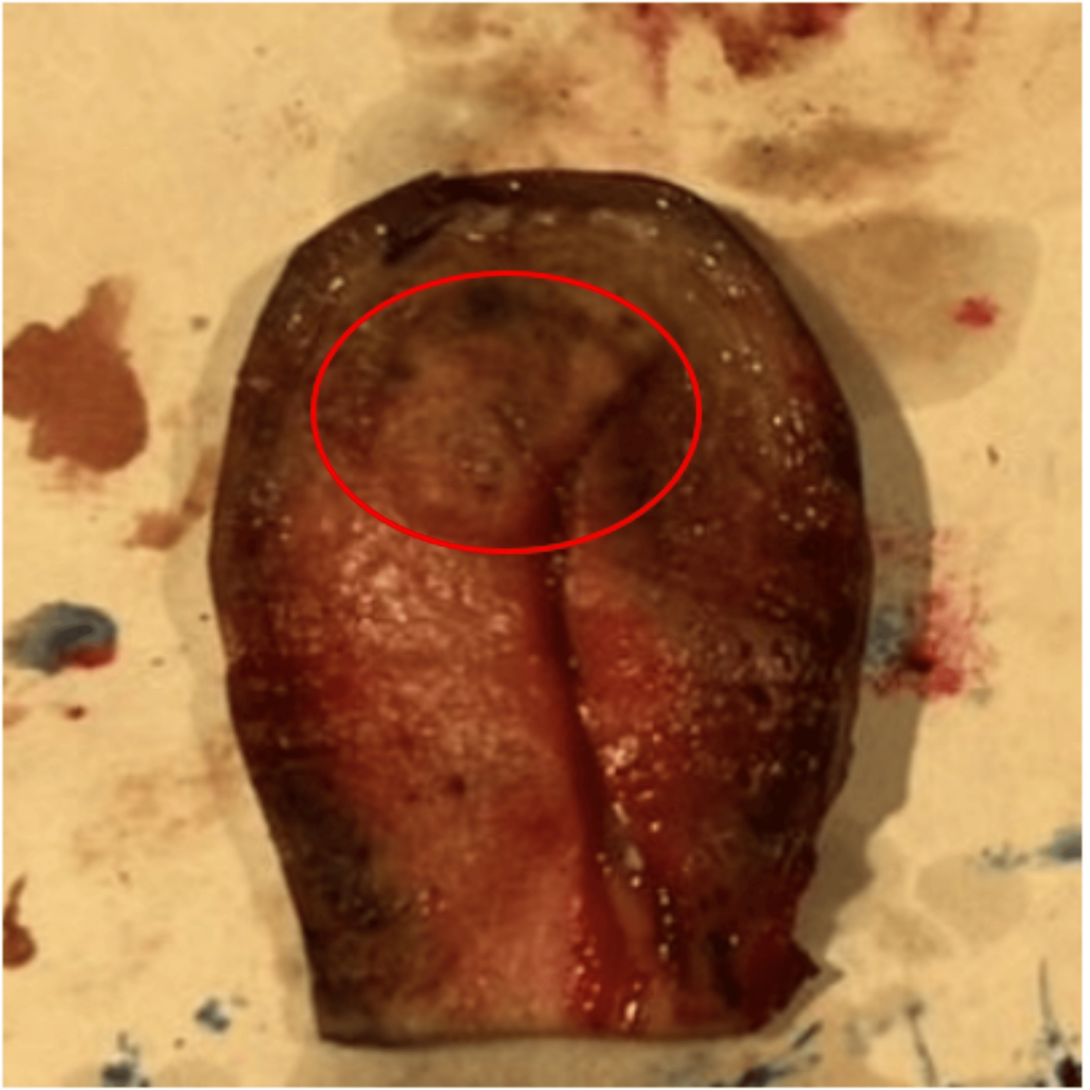
Macroscopic view showing a nodular lesion at the uterine fundus.

Macroscopic examination revealed a whitish lesion at the uterine fundus, with septations and a soft consistency (Figure 4).

**Figure 5:**
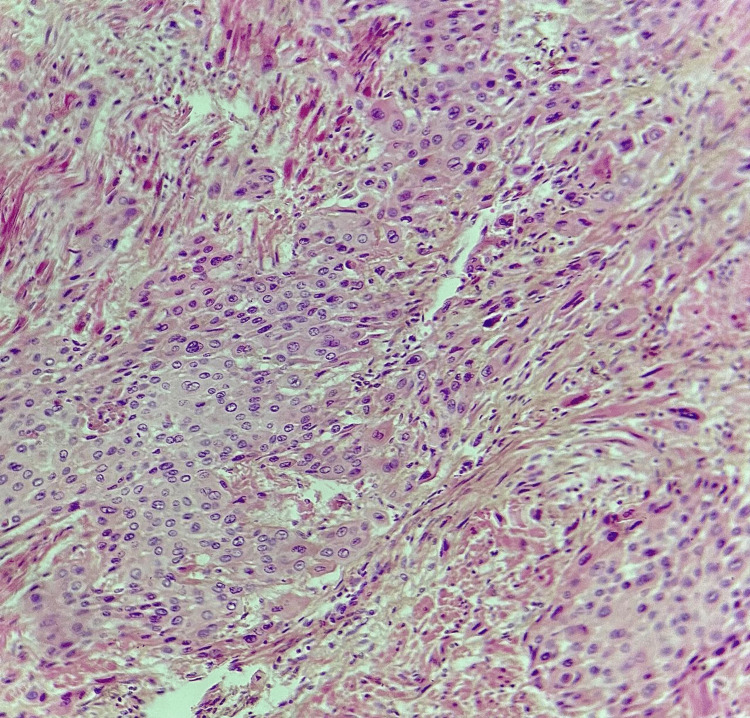
Histological image showing tumor proliferation arranged in clusters, cords, and nests (hematoxylin-eosin-saffron, x10).

**Figure 6:**
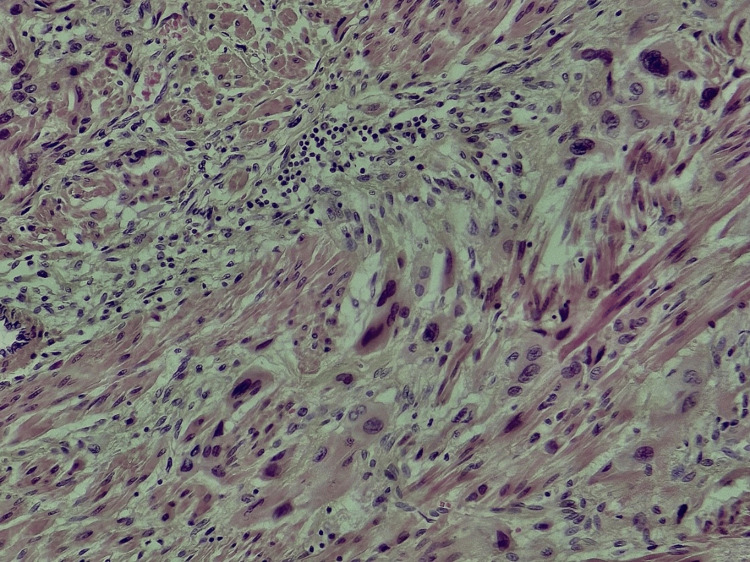
At the periphery, tumor cells infiltrate between the smooth muscle fibers (hematoxylin-eosin-saffron, x20).

Histological examination of the samples taken from the lesion revealed a well-defined tumor proliferation composed of intermediate trophoblastic-like cells arranged in clusters, cords, and nests (Figure 5). More peripherally, the cells become isolated, fusiform, and infiltrate between smooth muscle cells (Figure 6). The tumor cells were polygonal with nuclei that were typically round to ovoid, occasionally with irregular contours, and contained vesicular or hyperchromatic chromatin. They were surrounded by abundant cytoplasm that varied between amphophilic and eosinophilic staining.

**Figure 7:**
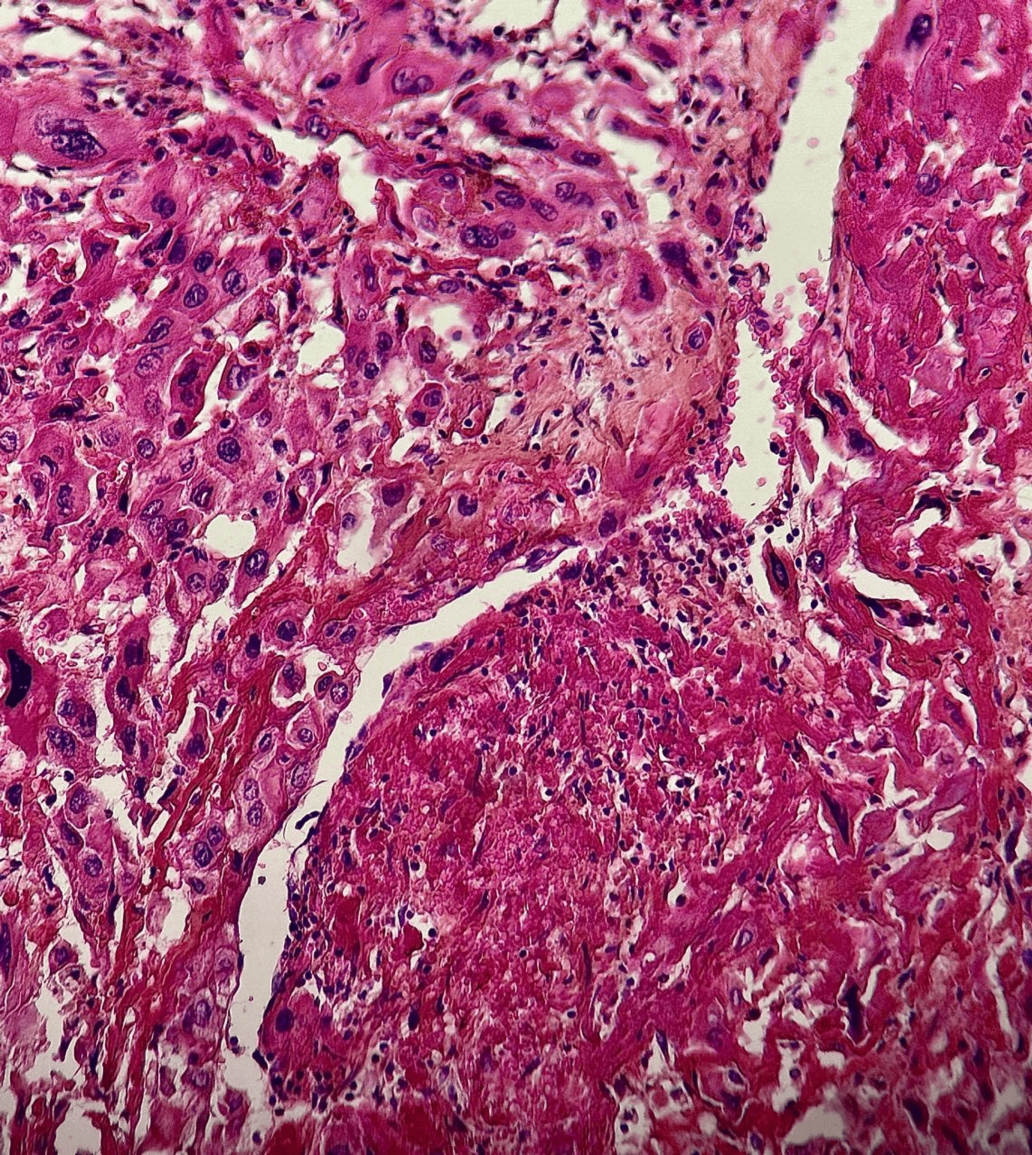
Tumor cells are polygonal with vesicular nuclei and eosinophilic cytoplasm, replacing the vascular walls in some areas with the presence of fibrinoid deposits surrounding the vascular structures (hematoxylin-eosin-saffron, x10).

Multinucleated cells were occasionally present. The tumor cells frequently wrapped around vascular structures, replacing the vessel wall, and were associated with eosinophilic fibrinoid deposits encircling the vessels (Figure 7).

Mitoses were observed at a rate of approximately five per 10 high-power fields (HPF).

**Figure 8:**
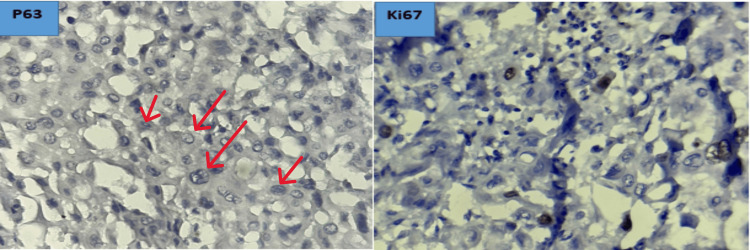
Immunohistochemical images showing the absence of anti-P63 labeling (red arrows) and an estimated tumor proliferation index of 10% (hematoxylin, x40).

An immunohistochemical study showed negative labeling of tumor cells with the anti-P63 antibody. The Ki-67 proliferation index of the tumor was estimated at 10% (Figure 8).

The patient is currently under regular monitoring of β-hCG levels, which remain negative to date.

## Discussion

PSTT affects women of childbearing age, typically between 32 and 35 years, and usually follows a normal pregnancy, though it can also occur after a molar pregnancy, spontaneous abortion, or ectopic pregnancy [2,3]. It often presents with amenorrhea or abnormal bleeding accompanied by abdominal enlargement, which may lead the patient to believe she is pregnant, as was the case with our patient [3]. The β-hCG level is typically low (<1000 mIU/L) [2,3]. PSTT can be diagnosed even after a few months to 20 years of the most recent pregnancy [6].

Through the clinical history and β-hCG levels, the pathologist can gain an initial orientation that may help in suspecting certain types of trophoblastic tumors while excluding others. Table 1 outlines the different clinical aspects of gestational trophoblastic tumors.

**Table 1:** Table comparing the clinical presentations of gestational trophoblastic tumors. GTD: gestational trophoblastic disease; PSTT: placental site trophoblastic tumor; ETT: epitheloid trophoblastic tumor; β-hCG: β-human chorionic gonadotropin. References [3,6].

	PSTT	ETT	Choriocarcinoma	Placental site nodule	Exaggerated placental site reaction
Age	20-63 years	15-66 years	29-31 years	Childbearing age	
Type of the last known pregnancy or GTD	Full-term pregnancy (2/3)/molar pregnancy/spontaneous abortion	Full-term pregnancy (67%)/spontaneous abortion/molar pregnancy	Hydatidiform mole (50%)/full-term pregnancy	Therapeutic abortion/cesarean delivery	Normal gestation/molar pregnancy
Latency	12-18 months	1-18 years	Weeks to several years	2-108 months	
Clinical presentation	History of missed abortion/abnormal uterine bleeding/amenorrhea/uterine enlargement	Abnormal uterine bleeding	Abnormal uterine bleeding/hemorrhage (at ectopic or metastatic sites)	Incidental findings	Asymptomatic/abnormal uterine bleeding
Serum β-hCG	Mild to moderate (<2000 UI/ml)	Mild to moderate (<2000 UI/ml)	High (>10000 UI/ml)	Normal levels	Normal levels

Macroscopically, PSTT appears as a distinct nodular mass, which may sometimes have a polypoid shape [3]. It typically measures between 1 and 10 cm, with a whitish-to-yellowish appearance on the cut surface [5]. In approximately 50% of cases, the mass invades the myometrium, reaches the serosa in 10%, and may occasionally extend to the broad ligament and adnexa [5,3]. Areas of hemorrhage and necrosis are observed in about 50% of cases [5].

Table 2 summarizes the different macroscopic features of gestational trophoblastic tumors.

**Table 2:** Table comparing the macroscopic characteristics of gestational trophoblastic tumors. PSTT: placental site trophoblastic tumor; ETT: epitheloid trophoblastic tumor; PSN: placental site nodule; APSN: atypical placental site nodule. References [3,5-9].

	PSTT	ETT	Choriocarcinoma	PSN /APSN	Exaggerated placental site reaction
Location	Uterine corpus/lower uterine segment. Extrauterine: very rare	Uterine corpus/lower uterine segment and cervix (50%)/fallopian tube/ovary/peritoneum	Uterus+++. Ectopic and metastatic locations have been reported: fallopian tube, ovary, cervix, lung, liver, kidney, and spleen	Uterus+++. Ectopic pregnancy locations (rare)	Uterus+++. Ectopic pregnancy locations (rare)
Shape	Nodular mass + well-circumscribed	Discrete nodular or cystic mass	Aggressive mass with an irregular contour	No lesion/solitary or multiple lesions	No macroscopic lesion
Consistency	Solid	Solid or cystic	Soft and friable	-	-
Color	White to yellow	Beige to brown	Dark red+++	Yellow, beige	-
Size	1-10 cm	0.5-4 cm	From microscopic to extensive mass+++	4-10 mm	-
Invasion	Deep myometrium (50%)/serosa (10%). Rarely board ligament and adnexa	Deep myometrium. Surrounding structures	Deep myometrium invasion/uterine perforation	Superficial myometrium	-
Hemorrhage	Focal	+++	+++	-	-
Necrosis	Focal (50%)	+++	+++	+	-

**Figure 9:**
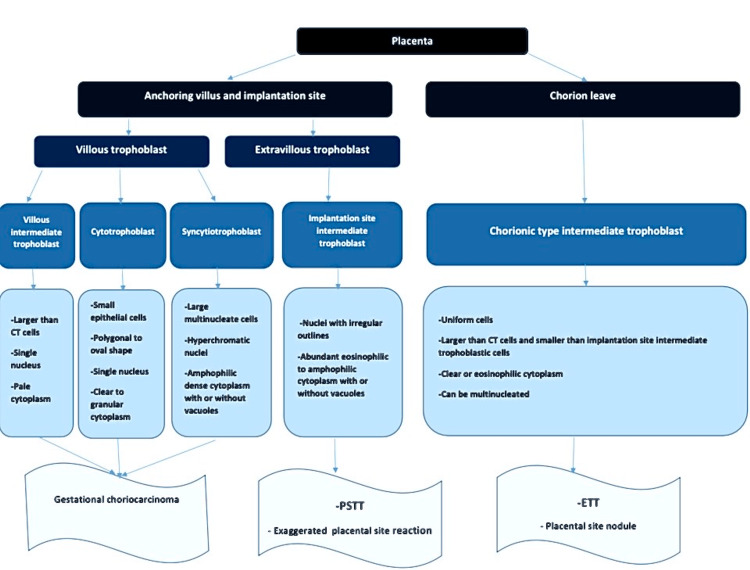
Histological characteristics of placental cells and origins of trophoblastic tumors. CT: cytotrophoblast; PSTT: placental site trophoblastic tumor; ETT: epitheloid trophoblastic tumor.

Gestational trophoblastic tumors differ in their cells of origin. Figure 9 summarizes the histological characteristics of the cells that compose the placenta and provides insight into the origins of various trophoblastic tumors [3,6].

The histological aspect is the cornerstone for differentiating gestational trophoblastic tumors. Table 3 helps compare the histological features of these tumors and allows for a systematic analysis.

**Table 3:** Table comparing the microscopic characteristics of gestational trophoblastic tumors. PSTT: placental site trophoblastic tumor; ETT: epitheloid trophoblastic tumor; PSN: placental site nodule; APSN: atypical placental site nodule; IT: intermediate trophoblasts; ST: syncytiotrophoblast; CT: cytotrophoblast; HPF: high-power field. * Vasculogenic mimicry: choriocarcinoma forms pseudovascular networks and blood lakes lined by trophoblastic cells, communicating with true vessels outside the tumor [3]. References [3,5,6,10].

	PSTT	ETT	Choriocarcinoma	PSN/APSN	Exaggerated placental site reaction
Architecture	Sheets+++	Nests/cords/masses	Compact masses of mononuclear cells surrounded by multinuclear cells	Clusters/cords/single cells	Cords/small nests
Surrounding tissue	Infiltration of endometrium and myometrium	Well-circumscribed +/- peripheric focal infiltration	Infiltration and destruction	Well-circumscribed	Infiltration of endometrium and myometrium with preserved architecture
Type of cells	Implantation site IT	Chorionic-type intermediate trophoblast	ST/CT/IT	Chorionic-type intermediate trophoblast	Implantation site IT
Nuclei	Hyperchromatic Irregular contours. Nuclear grooves/pseudo inclusions	Small and round/nucleoli+++	Pleomorphic/granular chromatin +/- nucleoli	Small and uniform++/+/-large, irregular, hyperchromatic +/-multinucleated cells	hyperchromatic + Irregular contours
Cytoplasm	Eosinophilic to amphophilic/clear	Granular eosinophilic/clear + well-defined cell membranes	Mononucleated cells: eosinophilic/clear. Multinucleated cells: markedly eosinophilic	Small cells: clear. Large cells: eosinophilic to amphophilic	Eosinophilic and abundant
Mitoses	2-4/10 HPF	0-9 mitoses/10 HPF	+++	Low mitotic activity in PSN <5 mm. Increased in PSN >5 mm.	Absent
Necrosis	-	+++	+++	-	-
Hemorrhage	+/-		+++	-	-
Vascular features	Infiltration of vessel walls with fibrinoid deposition	Central blood vessel in tumor nests +/- fibrinoid deposits	Vasculogenic mimicry*	-	infiltration of vessel walls
Calcifications	-	+++	-	-	-
Other characteristics	-	Re-epithelialization of endocervical/endometrial surfaces (specific). Eosinophilic hyaline-like material (resembles keratin). Decidualized stromal cells	-	Central hyalinized extracellular matrix	Chorionic villi are present

The cells of PSTT stain positively for pan-cytokeratin, epithelial membrane antigen (EMA), cytokeratin 18, placental alkaline phosphatase (PLAP), human leukocyte antigen-G (HLA-G) (diffuse staining), Mel-CAM (membranous staining), inhibin (focal), CD10, and HSD3B1, consistent with the general profile of trophoblastic cells [5,10]. They are negative for p63 and show diffuse and intense hPL staining, characteristic of the specific profile of implantation site intermediate trophoblasts (IT) [5,10]. β-hCG is detectable in rare syncytiotrophoblastic cells and some intermediate trophoblastic cells [10]. KI67 expression is estimated at 10-30% [6].

Each gestational trophoblastic tumor has an immunohistochemical panel that guides it toward its identification. Table 4 presents the various immunohistochemical markers of these tumors.

**Table 4:** Table comparing the immunohistochemical characteristics of gestational trophoblastic tumors. PSTT: placental site trophoblastic tumor; ETT: epitheloid trophoblastic tumor; PSN: placental site nodule; APSN: atypical placental site nodule; β-hCG: β-human chorionic gonadotropin; ST: syncytiotrophoblast; CK: cytokeratin; HPL: human placental lactogen. References [3,5,6,10,11].

	PSTT	ETT	Choriocarcinoma	PSN/APSN	Exaggerated placental site reaction
CK	Diffuse positivity	Diffuse positivity	Diffuse positivity	Diffuse positivity	Diffuse positivity
HSD3B1	Diffuse positivity	Diffuse positivity	Diffuse positivity	Diffuse positivity	Diffuse positivity
P63	Negative	Positive	Focally positive	Positive	Negative
HPL	Positive	Negative/focally positive	Focally positive	Negative/focally positive	Positive
β-hCG	Negative/focally positive (syncytiotrophoblast-like cells)	Negative/focally positive (<2%)	Diffuse positivity (ST)	Negative/focal and weak positivity	Negative/focally positive
Cyclin E		Diffuse positivity		Negative/focally positive	
Ki67	>10%	>12%	>50% of mononucleated cells	<8%	<1%

Unlike other trophoblastic tumors, PSTT is relatively resistant to chemotherapy, and surgery remains the main treatment option for patients with diseases confined to the uterus [2].

As reported by Lukinovic et al. [12], PSTT and ETT treatment is guided by two factors: an interval of ≥48 months from the causative pregnancy and stage IV disease. Stage I tumors (confined to the uterus) arising within <48 months are treated with total abdominal hysterectomy, along with retroperitoneal and pelvic lymphadenectomy if suspicious lymph nodes are present, with no need for adjuvant therapy. However, for tumors from a pregnancy >48 months ago or stage II-IV, aggressive platinum-based chemotherapy, including experimental options like high-dose chemotherapy or immunotherapy, is recommended and residual masses following treatment should be surgically removed [13]. Survival is approximately 100% for non-metastatic disease and 50-60% for metastatic disease [12].

The treatment of choriocarcinoma is based on chemotherapy, with a survival rate of 90% for non-metastatic forms and 70% for metastatic forms [10].

There is no targeted treatment for exaggerated placental site, though regular monitoring of β-hCG levels is required [14]. An exaggerated placental site is likely a physiological process that resolves after curettage and is not linked to a higher risk of persistent gestational trophoblastic disease [3].

The treatment of placental site nodules (PSNs) consists of the surgical excision of the nodule, and, for atypical PSNs, follow-up with pelvic MRI imaging is necessary [9].

**Figure 10:**
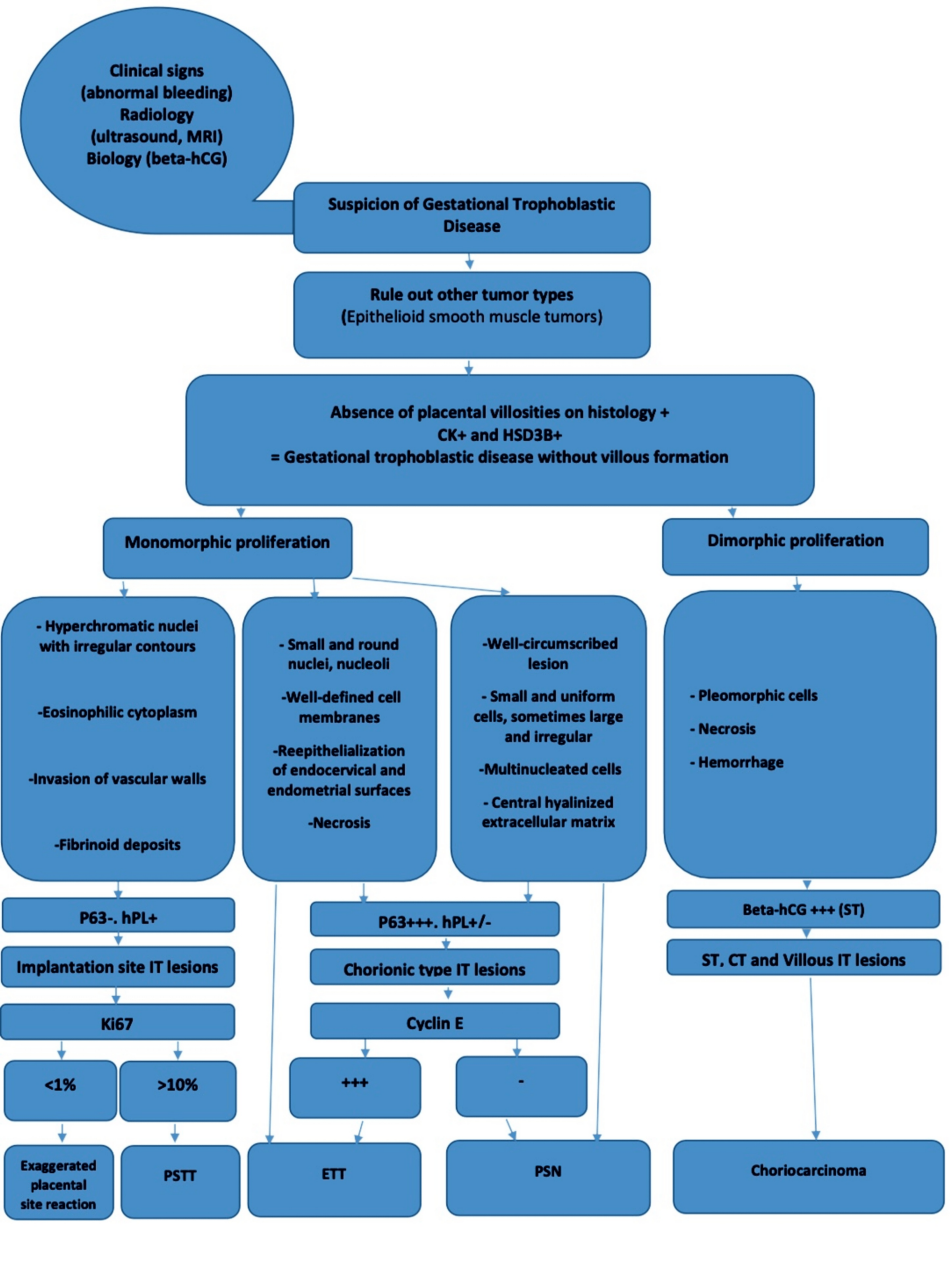
Methodology in pathological diagnosis of gestational trophoblastic disease. PSTT: placental site trophoblastic tumor; ETT: epitheloid trophoblastic tumor; PSN: placental site nodule; APSN: atypical placental site nodule; IT: intermediate trophoblasts; ST: syncytiotrophoblast; CT: cytotrophoblast; beta-hCG: beta-human chorionic gonadotropin.

Finally, Figure 10 summarizes the approach a pathologist may follow when gestational trophoblastic pathology is suspected [3,10].

## Conclusions

Gestational trophoblastic diseases are disorders that can occur following term pregnancies, miscarriages, or molar pregnancies. The pathologist plays a crucial role in distinguishing the type of disease, which is essential for therapeutic decision-making, surveillance, and prognosis prediction. This highlights the need for a thorough analysis combining clinical findings, radiology, gross examination, histology, and immunohistochemistry. Such an integrative approach is critical to ensure accurate diagnosis and appropriate management of these conditions.
